# Supplementary material for: A Multi-Trait Gaussian Kernel Genomic Prediction Model under Three Tunning Strategies
Source: Genes (Basel). 2022 Dec 3;13(12):2279. doi: 10.3390/genes13122279 (PMC9778253; doi:10.3390/genes13122279)
Supplement: Supplementary file 1 [file genes-13-02279-s001.zip › genes-2040773-SI.pdf]

SUPPLEMENTARY MATERIAL

RESULTS

Dataset 4 Cotton

The NRMSE of traits LEN and STR were denoted as the NRMSE_LEN and NRMSE_STR respectively. As seen in **Table S1**, in terms of NRMSE for the LEN trait, the best performance under BO strategy was in environments BK (0.859) and CO (0.813), while under the GsR strategy it was in environment SG (0.855), and under the NT strategy in environments BR (0.895), DD (0.914), EM (0.829), MV (0.832) and Global (0.788).

For the STR trait, the best predictions (lower NRMSE) were observed under the GrS strategy [BK (0.63), BR (0.55), CO (0.807), DD (0.986), EM (0.601), MV (0.691), SG (0.558)], with the exception in Global (across environments) where the lowest NRMSE was 0.663 under the BO strategy. More details are provided in **Table S1**. Standard error of prediction performance for every environment and across environments (Global) are provided in **Table S2**.

Across traits the prediction performance can be observed in **Figure S1A**, where the best predictions (lower NRMS) can be found under the BO and GrS strategies and the worst under the NT strategy. Across traits we can observe that the RE of comparing the NT strategy versus BO strategy for each environment and across environments were 1.022 (BK), 1.016 (BR), 1.019 (CO), 1.007 (DD), 1.035 (EM), 1.028 (MV), 1.05 (SG) and 1.019 (Global) (Figure S1_B). This indicates that the BO method outperformed NT strategy in terms of NRMSE in all environments by 2.2% (BK), 1.6% (BR), 1.9% (CO), 0.7% (DD), 3.5% (EM), 2.8% (MV), 5% (SG) and 1.9% (Global). While the RE of comparing the NT strategy versus GrS strategy for each environment and across environments was 1.02 (BK), 1.02 (BR), 1.008 (CO), 1.009 (DD), 1.044 (EM), 1.03 (MV), 1.06 (SG) and 1.021 (Global) (**Figure S1B**). This indicates that the GrS method outperformed NT strategy in terms of NRMSE in all the environments mentioned by 2.0% (BK), 2.0% (BR), 0.8% (CO), 0.9% (DD), 4.4% (EM), 3% (MV),6% (SG) and 2.1% (Global). Finally, the RE of comparing the GrS method versus the BO method was 1.002 (BK), 0.996 (BR), 1.01 (CO), 0.998 (DD), 0.997 (EM), 0.997 (MV), 0.99 (SG)

and 0.997 (Global) (**Figure S1B**). This means that the BO strategy is slightly worst in terms of prediction performance than the GrS method since the RE in most cases was less than one. For details see **Table S1**.

The prediction performance in terms of NRMSE of each trait across environments are given in **Figure S1C** and the relative efficiencies of comparing NT versus BO, NT versus GrS and GrS and BO are given in **Figure S1D**, where we BO and GrS were the best strategies for tuning and NT the worst. Also, no relevant differences between the BO and GrS methods were observed.

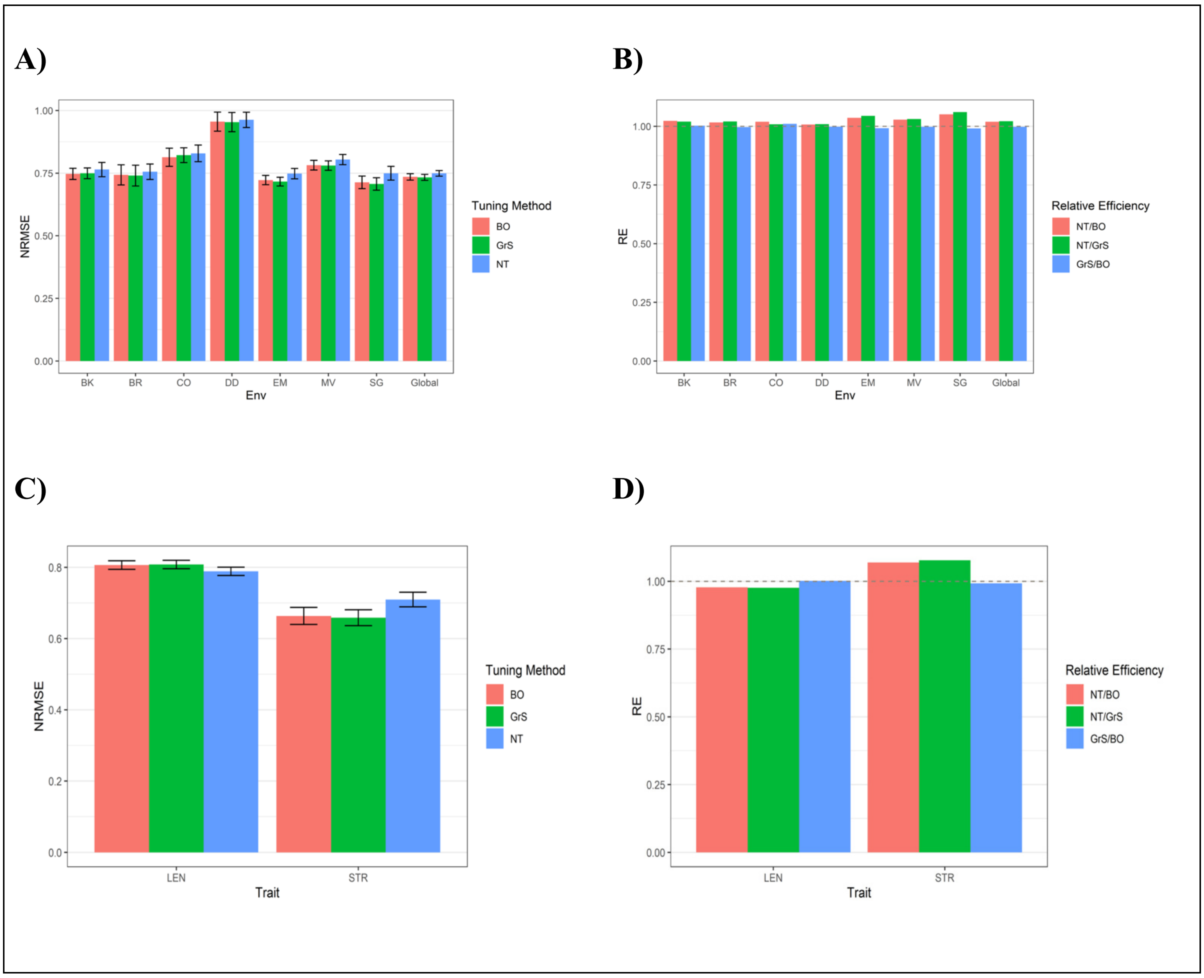

Figure S1. Data set 4 Cotton. A) Prediction performance in terms of Normalized root mean squared error (NRMSE) for each environment (BK, BR, CO, DD, EM, MV and SG), across environments (Global) and across traits (LEN and STR) with three tuning strategies (BO, GrS and NT) under 7 Fold Cross-Validation (7FCV). **B**) Relative efficiency for each environment (BK, BR, CO, DD, EM, MV and SG), across environments (Global) and across traits with three tuning strategies (BO, GrS and NT) under 7-Fold Cross-Validation (7FCV). **C**) Prediction performance in terms of Normalized root mean squared error (NRMSE) for each trait (LEN and STR) across environments with three tuning strategies (BO, GrS and NT) under 7 Fold Cross-Validation (7FCV). **D**) Relative efficiency for each trait (LEN and STR) across environments with three tuning strategies (BO, GrS and NT) under 7 Fold Cross-Validation (7FCV). When RE > 1 the denominator method outperforms the numerator in terms of prediction performance.

**Table S1.** Prediction performance for every environment and across environments (Global) of the **dataset 4 (Cotton)** in terms of Normalized Root Mean Square Error (NRMSE) under three tuning methods (BO, GrS and NT) for two cotton traits (LEN and STR) data set. RE denotes relative efficiency. The RE in rows corresponding to BO were computed dividing the NRMSE under NT by the NRMSE under BO. While the RE in rows corresponding to GrS were computed dividing the NRMSE under NT by the NRMSE under GrS. While those RE in the rows corresponding to NT strategy were computed dividing the NRMSE under GrS by the NRMSE under BO					
Tuning Type	Environment	NRMSE_LEN	NRMSE_STR	NRMSE	RE
Bayesian Optimization	BK	0.8598	0.6343	0.74705	1.0228900
Bayesian Optimization	BR	0.9241	0.5620	0.74305	1.0165534
Bayesian Optimization	CO	0.8131	0.8136	0.81335	1.0190570
Bayesian Optimization	DD	0.9240	0.9867	0.95535	1.0076935
Bayesian Optimization	EM	0.8324	0.6120	0.72220	1.0358626
Bayesian Optimization	MV	0.8652	0.6984	0.78180	1.0283960
Bayesian Optimization	SG	0.8554	0.5709	0.71315	1.0507607
Bayesian Optimization	Global	0.8064	0.6634	0.73490	1.0192543
Grid Search	BK	0.8676	0.6306	0.74910	1.0200908
Grid Search	BR	0.9301	0.5504	0.74025	1.0203985
Grid Search	CO	0.8357	0.8076	0.82165	1.0087629
Grid Search	DD	0.9211	0.9862	0.95365	1.0094899
Grid Search	EM	0.8305	0.6019	0.71620	1.0445406
Grid Search	MV	0.8683	0.6917	0.78000	1.0307692
Grid Search	SG	0.8552	0.5582	0.70670	1.0603509

Table S1. Prediction performance for every environment and across environments (Global) of the **dataset 4 (Cotton)** in terms of Normalized Root Mean Square Error (NRMSE) under three tuning methods (BO, GrS and NT) for two cotton traits (LEN and STR) data set. RE denotes relative efficiency. The RE in rows corresponding to BO were computed dividing the NRMSE under NT by the NRMSE under BO. While the RE in rows corresponding to GrS were computed dividing the NRMSE under NT by the NRMSE under GrS. While those RE in the rows corresponding to NT strategy were computed dividing the NRMSE under GrS by the NRMSE under BO

Tuning Type	Environment	NRMSE_LEN	NRMSE_STR	NRMSE	RE
Grid Search	Global	0.8078	0.6585	0.73315	1.0216872
NoTuning	BK	0.8623	0.6660	0.76415	1.0027441
NoTuning	BR	0.8953	0.6154	0.75535	0.9962317
NoTuning	CO	0.8254	0.8323	0.82885	1.0102047
NoTuning	DD	0.9149	1.0105	0.96270	0.9982205
NoTuning	EM	0.8299	0.6663	0.74810	0.9916921
NoTuning	MV	0.8321	0.7759	0.80400	0.9976976
NoTuning	SG	0.8689	0.6298	0.74935	0.9909556
NoTuning	Global	0.7886	0.7095	0.74905	0.9976187

**Table S2.** Standard error of prediction performance for every environment and across environments (Global) of two traits (LEN and STR) for the **dataset 4 (Cotton)** in terms of Normalized Root Mean Square Error (NRMSE) under three methods of tuning (BO, GrS and NT). RE denotes relative efficiency.				
Tuning Type	Environment	NRMSE_SE_LEN	NRMSE_SE_STR	NRMSE_SE
Bayesian Optimization	BK	0.0168	0.0458	0.015650
Bayesian Optimization	BR	0.0878	0.0263	0.028525
Bayesian Optimization	CO	0.0301	0.0726	0.025675
Bayesian Optimization	DD	0.0205	0.0875	0.027000
Bayesian Optimization	EM	0.0168	0.0354	0.013050
Bayesian Optimization	MV	0.0144	0.0405	0.013725
Bayesian Optimization	SG	0.0256	0.0452	0.017700
Bayesian Optimization	Global	0.0120	0.0238	0.008950
Grid Search	BK	0.0147	0.0461	0.015200
Grid Search	BR	0.0890	0.0285	0.029375
Grid Search	CO	0.0156	0.0681	0.020925
Grid Search	DD	0.0184	0.0904	0.027200
Grid Search	EM	0.0173	0.0320	0.012325
Grid Search	MV	0.0164	0.0370	0.013350
Grid Search	SG	0.0264	0.0436	0.017500
Grid Search	Global	0.0117	0.0226	0.008575
No Tuning	BK	0.0327	0.0481	0.020200

**Table S2.** Standard error of prediction performance for every environment and across environments (Global) of two traits (LEN and STR) for the **dataset 4 (Cotton)** in terms of Normalized Root Mean Square Error (NRMSE) under three methods of tuning (BO, GrS and NT). RE denotes relative efficiency.				
Tuning Type	Environment	NRMSE_SE_LEN	NRMSE_SE_STR	NRMSE_SE
No Tuning	BR	0.0655	0.0218	0.021825
No Tuning	CO	0.0155	0.0787	0.023550
No Tuning	DD	0.0246	0.0617	0.021575
No Tuning	EM	0.0277	0.0304	0.014525
No Tuning	MV	0.0125	0.0448	0.014325
No Tuning	SG	0.0308	0.0468	0.019400
No Tuning	Global	0.0116	0.0206	0.008050

Dataset 5 Disease

Here we denote NRMSE_PTR, NRMSE_SB and NRMSE_SN the NRMSE of traits PTR, SB and SN respectively. As shown in **Table S3**. in terms of NRMSE for the PTR trait, the best performance under the GrS strategy was observed in environments Env1 (0.857), Env4 (0.853), Env5 (0.854), Env6 (0.863) and Global (0.826), while the environment with the best NRMSE under the BO strategy was Env2 (0.867) and under NT strategy was Env3 (0.844). For trait SB, the best NRMSE values were observed under the GrS strategy in environments Env1 (0.844), Env2 (0.838), Env3 (0.811), Env4 (0.857), Env5 (0.849), Env6 (0.846) and Global (0.817). For trait SN, the best predictions (lower NRMSE) were observed under the GrS strategy [Env1 (0.763), Env3 (0.758), Env4 (0.69), Env5 (0.796) and Global (0.712)], except in the case of Env2 and Env6 were best NRMSE values were 0.76 and 0.737 under the BO strategy. Standard error of prediction performance for every environment and across environments (Global) are provided in **Table S4**.

Summarizing across traits for each environment shows in **Figure S2A** that the best predictions (lower NRMS) were observed under the BO and GrS strategies and the worst

under the NT strategy. Across traits we can also observe that the RE of comparing the NT strategy versus BO strategy for each environment and across environments were 1.021 (Env1), 1.023 (Env2), 1.024 (Env3), 1.02 (Env4), 1.022 (Env5), 1.033 (Env6) and 1.023 (Global) (**Figure S2B**). This indicates that the BO method outperformed NT strategy in terms of NRMSE in all environments by 2.1% (Env1), 2.3% (Env2), 2.4% (Env3), 2% (Env4), 2.2% (Env5), 3.3% (Env6) and 2.3% (Global). While the RE of comparing the NT strategy versus GrS strategy for each environment and across environments were 1.023 (Env1), 1.025 (Env2), 1.024 (Env3), 1.026 (Env4), 1.039 (Env5), 1.029 (Env6) and 1.027 (Global) (Figure S2_B). This indicates that the GrS method outperformed the NT strategy in terms of NRMSE in all the environments mentioned by 2.3% (Env1), 2.5% (Env2), 2.4% (Env3), 2.6% (Env4), 3.9% (Env5), 2.9% (Env6) and 2.7% (Global). Finally, the RE of comparing the GrS method versus the BO method were 0.996 (Env1), 1.000 (Env2), 0.996 (Env3), 0.995 (Env4), 0.994 (Env5), 0.995 (Env6) and 0.996 (Global) (**Figure S2B**). This means that the BO strategy is slightly worse in terms of prediction performance than the GrS method, since the RE in most case was slightly less than one. For more details, see **Table S3**.

The prediction performance in terms of NRMSE of each trait across environments are given in **Figure S2C** and the relative efficiencies of comparing NT versus BO, NT versus GrS and GrS and BO are given in **Figure S2D**, where in all three traits, the tuning strategies of BO and GrS were slightly better than the NT strategy.

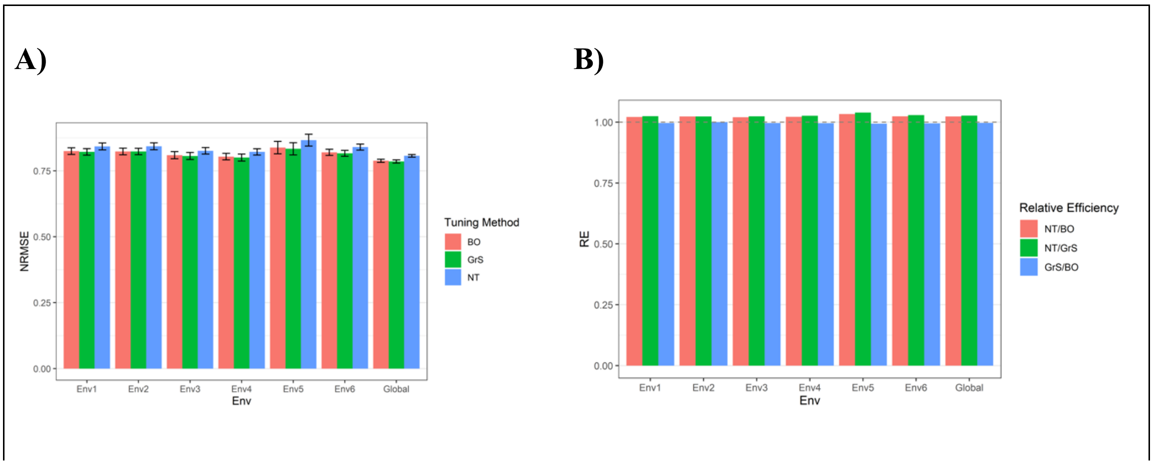

C)

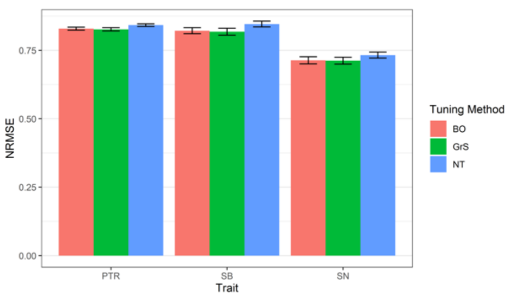

D)

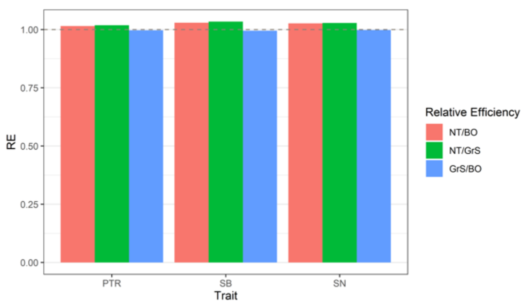

Figure S2. A) Prediction performance for **Dataset 5 (Disease)** in terms of Normalized root mean squared error (NRMSE) for each environment (Env1, Env2, Env3, Env4, Env5, Env6), across environments (Global) and across traits with three tuning strategies (BO, GrS and NT) under 7-fold cross-validation (7FCV). **B**) Relative efficiency for each environment (Env1, Env2, Env3, Env4, Env5, Env6), across environments (Global) and across traits (PTR, SB and SN) with three tuning strategies (BO, GrS and NT) under 7FCV. **C**) Prediction performance in terms of Normalized root mean squared error (NRMSE) for each trait (PRT, SB and SN) across environments with three tuning strategies (BO, GrS and NT) under 7-fold cross-validation (7FCV). **D**) Relative efficiency for each trait (PRT, SB and SN) across environments with three tuning strategies (PRT, SB and SN) under 7-fold cross-validation (7FCV). When RE > 1 the denominator method outperforms the numerator in terms of prediction performance.

Table S3. Prediction performance for every environment and across environments (Global) of the **Dataset 5** (**Disease**) in terms of Normalized Root Mean Square Error (NRMSE) under three tuning methods (BO, GrS and NT) and for 3 traits (SB, SN, SE). RE denotes relative efficiency. The RE in rows corresponding to BO were computed dividing the NRMSE under NT by the NRMSE under BO. While the RE in rows corresponding to GrS were computed dividing the NRMSE under NT by the NRMSE under GrS. While those RE in the rows corresponding to NT strategy were computed dividing the NRMSE under GrS by the NRMSE under BO.

Tuning Type	Environment	NRMSE_PTR	NRMSE_SB	NRMSE_SN	NRMSE	RE
Bayesian Optimization	Env1	0.8593	0.8490	0.7660	0.8247667	1.0213394
Bayesian Optimization	Env2	0.8676	0.8415	0.7602	0.8231000	1.0239744
Bayesian Optimization	Env3	0.8483	0.8206	0.7589	0.8092667	1.0202653
Bayesian Optimization	Env4	0.8548	0.8627	0.6939	0.8038000	1.0222278
Bayesian Optimization	Env5	0.8586	0.8527	0.8030	0.8381000	1.0336475
Bayesian Optimization	Env6	0.8732	0.8490	0.7374	0.8198667	1.0244755
Bayesian Optimization	Gbal	0.8292	0.8216	0.7134	0.7880667	1.0237290
Grid Search	Env1	0.8571	0.8446	0.7636	0.8217667	1.0250679
Grid Search	Env2	0.8689	0.8389	0.7617	0.8231667	1.0238915
Grid Search	Env3	0.8484	0.8116	0.7585	0.8061667	1.0241885
Grid Search	Env4	0.8530	0.8574	0.6906	0.8003333	1.0266556
Grid Search	Env5	0.8542	0.8493	0.7968	0.8334333	1.0394353
Grid Search	Env6	0.8635	0.8468	0.7383	0.8162000	1.0290778
Grid Search	Global	0.8266	0.8178	0.7121	0.7855000	1.0270741
No Tuning	Env1	0.8780	0.8695	0.7796	0.8423667	0.9963626

**Table S3.** Prediction performance for every environment and across environments (Global) of the **Dataset 5** (**Disease**) in terms of Normalized Root Mean Square Error (NRMSE) under three tuning methods (BO, GrS and NT) and for 3 traits (SB, SN, SE). RE denotes relative efficiency. The RE in rows corresponding to BO were computed dividing the NRMSE under NT by the NRMSE under BO. While the RE in rows corresponding to GrS were computed dividing the NRMSE under NT by the NRMSE under GrS. While those RE in the rows corresponding to NT strategy were computed dividing the NRMSE under GrS by the NRMSE under BO.						
Tuning Type	Environment	NRMSE_PTR	NRMSE_SB	NRMSE_SN	NRMSE	RE
No Tuning	Env2	0.8881	0.8694	0.7710	0.8428333	1.0000810
No Tuning	Env3	0.8440	0.8614	0.7716	0.8256667	0.9961694
No Tuning	Env4	0.8656	0.8841	0.7153	0.8216667	0.9956872
NoTuning	Env5	0.8895	0.8716	0.8378	0.8663000	0.9944318
No Tuning	Env6	0.8857	0.8775	0.7566	0.8399333	0.9955277
No Tuning	Global	0.8420	0.8459	0.7324	0.8067667	0.9967431

**Table S4.** Standard error of prediction performance for every environment and across environments (Global) of the **dataset 5 (Disease)** in terms of Normalized Root Mean Square Error (NRMSE) under three methods of tuning (BO, GrS and NT) for three traits (PTR, SB, SN). RE denotes relative efficiency.					
Tuning Type	Environment	NRMSE_SE_PTR	NRMSE_SE_SB	NRMSE_SE_SN	NRMSE_SE
Bayesian Optimization	Env1	0.0155	0.0311	0.0187	0.0125670
Bayesian Optimization	Env2	0.0216	0.0166	0.0275	0.0126440
Bayesian Optimization	Env3	0.0158	0.0201	0.0342	0.0134908
Bayesian Optimization	Env4	0.0196	0.0266	0.0187	0.0124900
Bayesian Optimization	Env5	0.0277	0.0321	0.0624	0.0235174

Table S4. Standard error of prediction performance for every environment and across environments (Global) of the **dataset 5 (Disease)** in terms of Normalized Root Mean Square Error (NRMSE) under three methods of tuning (BO, GrS and NT) for three traits (PTR, SB, SN). RE denotes relative efficiency.

Tuning Type	Environment	NRMSE_SE_PTR	NRMSE_SE_SB	NRMSE_SE_SN	NRMSE_SE
Bayesian Optimization	Env6	0.0271	0.0129	0.0210	0.0117395
Bayesian Optimization	Global	0.0055	0.0111	0.0129	0.0056773
Grid Search	Env1	0.0147	0.0306	0.0186	0.0122976
Grid Search	Env2	0.0213	0.0147	0.0268	0.0120859
Grid Search	Env3	0.0161	0.0191	0.0334	0.0132021
GridSearch	Env4	0.0203	0.0274	0.0211	0.0132406
Grid Search	Env5	0.0278	0.0323	0.0596	0.0230363
GridSearch	Env6	0.0248	0.0121	0.0218	0.0112968
Grid Search	Global	0.0061	0.0126	0.0128	0.0060622
No Tuning	Env1	0.0184	0.0297	0.0195	0.0130096
No Tuning	Env2	0.0239	0.0135	0.0301	0.0129904
No Tuning	Env3	0.0173	0.0196	0.0274	0.0123745
No Tuning	Env4	0.0169	0.0267	0.0179	0.0118357
No Tuning	Env5	0.0277	0.0342	0.0538	0.0222665
No Tuning	Env6	0.0278	0.0097	0.0214	0.0113353
No Tuning	Global	0.0048	0.0106	0.0110	0.0050807
